# Relationship between Exposure to Vector Bites and Antibody Responses to Mosquito Salivary Gland Extracts

**DOI:** 10.1371/journal.pone.0029107

**Published:** 2011-12-14

**Authors:** Albin Fontaine, Aurélie Pascual, Eve Orlandi-Pradines, Ibrahima Diouf, Franck Remoué, Frédéric Pagès, Thierry Fusaï, Christophe Rogier, Lionel Almeras

**Affiliations:** 1 Unité de Parasitologie, Antenne Marseille de l'Institut de Recherche Biomédicale des Armées (IRBA), Marseille, France; 2 Unité d'Entomologie Médicale, Antenne Marseille de l'Institut de Recherche Biomédicale des Armées (IRBA), Marseille, France; 3 Caractérisation des Populations de vecteurs, Institut de Recherche pour le Développement (IRD), Montpellier, France; 4 Institut Pasteur de Madagascar, Antananarivo, Madagascar; University of Lausanne, Switzerland

## Abstract

Mosquito-borne diseases are major health problems worldwide. Serological responses to mosquito saliva proteins may be useful in estimating individual exposure to bites from mosquitoes transmitting these diseases. However, the relationships between the levels of these IgG responses and mosquito density as well as IgG response specificity at the genus and/or species level need to be clarified prior to develop new immunological markers to assess human/vector contact. To this end, a kinetic study of antibody levels against several mosquito salivary gland extracts from southeastern French individuals living in three areas with distinct ecological environments and, by implication, distinct *Aedes caspius* mosquito densities were compared using ELISA. A positive association was observed between the average levels of IgG responses against *Ae. caspius* salivary gland extracts and spatial *Ae. caspius* densities. Additionally, the average level of IgG responses increased significantly during the peak exposure to *Ae. caspius* at each site and returned to baseline four months later, suggesting short-lived IgG responses. The species-specificity of IgG antibody responses was determined by testing antibody responses to salivary gland extracts from *Cx. pipiens*, a mosquito that is present at these three sites at different density levels, and from two other *Aedes* species not present in the study area (*Ae. aegypti* and *Ae. albopictus*). The IgG responses observed against these mosquito salivary gland extracts contrasted with those observed against *Ae. caspius* salivary gland extracts, supporting the existence of species-specific serological responses. By considering different populations and densities of mosquitoes linked to environmental factors, this study shows, for the first time, that specific IgG antibody responses against *Ae. caspius* salivary gland extracts may be related to the seasonal and geographical variations in *Ae. caspius* density. Characterisation of such immunological-markers may allow the evaluation of the effectiveness of vector-control strategies or estimation of the risk of vector-borne disease transmission.

## Introduction

Mosquito-borne diseases are a major health problem worldwide, and cause important morbidity and mortality in tropical areas [1], [2]. To a lesser extend, the European population is also exposed to a variety of mosquito-borne pathogens. Outbreaks of mosquito-borne diseases with significant human health implications occurred on a mass scale in Europe in the last century. These outbreaks included the following: Dengue virus in Greece in 1928 [3], West Nile virus in Camargue in 1962 [4] and in Romania in 1996 [5], Sindbis virus in Finland in 2002 [6] and more recently, Chikungunya virus in Italy [7].

Numerous indexes can provide a comprehensive understanding of the potential for mosquito-borne disease transmission and the dynamics of these diseases in human populations, such as the vectorial capacity, the basic reproductive rate (R0) and the entomological inoculation rate (EIR) [8], [9]. These indicators of disease transmission levels can also quantify the impact of vector-control strategies [9]. These indexes mainly depend on entomological parameters that can be measured in the field, including the human-biting rate (HBR) (average number of bites per individual per day received from a mosquito species) [10]. This parameter is currently estimated by entomological methods, such as human landing catches, or other strategies based on attractant traps (e.g., light traps, carbon-dioxide traps, odour-baited traps) [11]. These entomological methods have proven efficacy for monitoring the density of mosquitoes relative to the density of the human population [12]–[14] but the HBR has been shown to vary within small geographic areas [15], [16], meaning that the results of local catches cannot be extrapolated to larger areas. These methods are not adapted to consider differences found within a population, which include differential attractiveness to mosquitoes [17] or other environmental and socioeconomic factors that could induce important variations in individual exposure to vector bites. Moreover, entomological methods can be labour intensive, expensive and difficult to implement when mosquito numbers are low or because of logistical constraints. In addition, the deliberate exposure of human volunteers to vectors has raised some ethical issues related to human landing catches, which remains the most reliable method to estimate host/vector contacts [11].

The use of immunologically based techniques to estimate individual exposure to arthropod vector bites, such as those from mosquitoes, ticks, sand flies and *Glossina*, has been described in several studies [18]–[22]. The saliva of hematophagous arthropods contains a complex mixture of biologically active proteins. These proteins may modify hemostatic responses and induce both cellular immunity and the production of specific antibodies [23], [24]. As described previously, mosquito salivary gland extracts can induce an IgG antibody response in individuals living in endemic areas [25]–[27] and in travellers transiently exposed to vectors in tropical areas [28], suggesting that salivary proteins can potentially be used as immunological markers to evaluate individual exposure to mosquito bites.

Mosquito densities and species diversity can be influenced by the surrounding landscape, even in restricted areas [29], [30]. The Mediterranean coast of southern France presents areas with distinct demographic and ecological conditions, ranging from large wetland areas in the Rhone River delta (Camargue) to highly urbanised environments (city of Marseille). These contrasting landscapes mirror the density and geographical spread of some mosquito species, notably *Aedes caspius*.

*Ae. caspius* is a Paleartic species that has demonstrated the ability to transmit the Rift Valley fever virus and the Chikungunya virus in the laboratory [31], [32]. This mosquito species was also suspected to be involved in the 1993 Rift Valley fever outbreak in Egypt [33]. Despite its low vector competence for these viruses, *Ae. caspius* should be considered a potential vector in wetland areas due to its high anthropophily and its abundance. *Aedes caspius* is well adapted to swampy environments: it tolerates varying levels of salinity in larval breeding sites, and its larval development is linked to the alternating dry and wet seasons in areas where its eggs are laid [34]. After abundant rainfalls events, a massive, synchronous adult population emerges and becomes a nuisance [35]. In Camargue, *Ae. caspius* is active from March to November [36], [37]. *Ae. aegypti* and *Ae. albopictus* mosquitoes, both vectors of arboviruses (*e.g.*, yellow fever, dengue or chikungunya viruses), were not endemic in the study area at the time of the present work [38].

To assess whether exposure to different densities and/or species of mosquitoes throughout the year could influence the antibody response against mosquito salivary gland extracts, we tested the IgG response to *Ae. caspius* salivary gland extracts of individuals living in three southern French areas (Camargue, Fos-sur-mer and Marseille) with distinct ecological environments at three time points (February 2007, September 2007 and January 2008). We concomitantly evaluated the IgG responses to salivary gland extracts from *Culex pipiens, Aedes albopictus* and *Aedes aegypti* as controls. The temporal and spatial evolution of IgG responses according to mosquito species will be discussed.

## Results

### Kinetics of IgG antibody responses against Ae. caspius salivary gland extracts (AecSGE) from individuals living in distinct ecological environments

**Figure 1:**
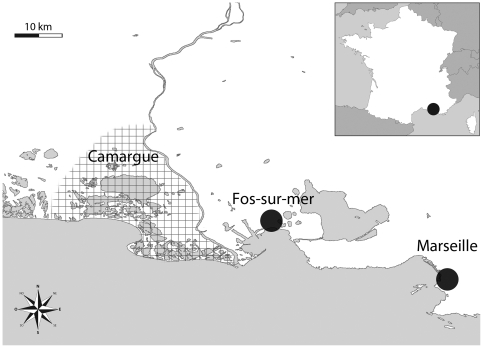
Location of the study sites. The city of Marseille and the town of Fos-sur-mer are indicated by circles, and the Camargue area is cross-hatched.

**Figure 2:**
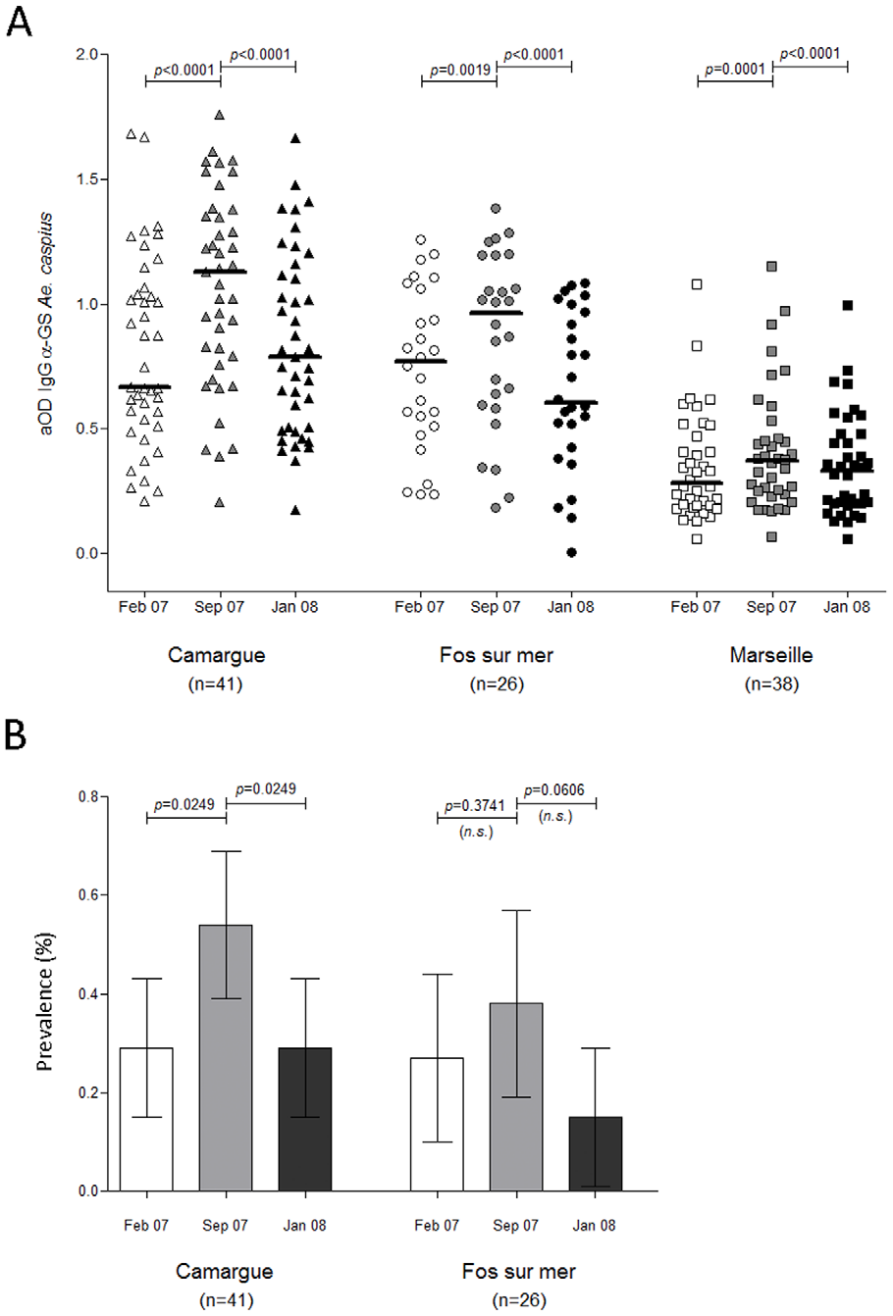
Spatial and seasonal variations of IgG responses to *Ae. caspius* salivary gland protein extracts. (A) IgG serum levels against *Ae. caspius* salivary gland protein extracts. Scatter plots of IgG antibody responses against *Ae. caspius* salivary gland extracts in individuals from Marseille (squares), Fos-sur-mer (circles) and Camargue (triangles) at three times February 07 (T1), September 07 (T2) and January 08 (T3), are presented with the sampling time indicated in white, grey and black symbols, respectively. Antibody responses are represented by aOD: the mean OD value of wells with antigen minus the mean OD value of wells without antigen. Each point shows the aOD value for a single individual. Horizontal bars show medians. Differences between the two time points at a single site were tested using the Wilcoxon signed-rank test. *p*-values are indicated. (B) Seasonal variation of seroprevalence against *Ae. caspius* salivary gland protein extracts at the three study sites. Seroprevalence against *Ae. caspius* salivary gland extracts in individuals from Fos-sur-mer and Camargue at three times February 07 (T1, white bars), September 07 (T2, light grey bars) and January 08 (T3, dark grey bars) are represented. Whiskers indicate the 95% confidence interval. The *p* values were determined by the chi-squared test.

First, we determined whether mosquito density, linked to the ecological environment and season, could influence the IgG responses against mosquito salivary gland extracts. Thus, the IgG responses against *AecSGE* were assessed in individuals living in Camargue, Fos-sur-mer or Marseille (Figure 1) at three time points: February 2007 (T1) and January 2008 (T3), which corresponded to periods outside of the *Ae. caspius* exposure peak, and September 2007 (T2), which corresponded to the *Ae. caspius* exposure peak period [36]. Independent of the sampling time (i.e., T1, T2 or T3), the IgG antibody responses against *Aec*SGE were significantly different among the sites (Kruskal-Wallis test, *p*<0.0001, Table S1). Additionally, independent of the site, the IgG response against *Aec*SGE increased significantly from T1 to T2 (Figure 2A, Wilcoxon matched pairs test, *p*<0.0001, p<0.0018 and p<0.0001 for the Camargue, Fos-sur-mer and Marseille sites, respectively, Table S1) and decreased significantly from T2 to T3 (Wilcoxon matched pairs test, *p*<0.0001 for the three sites). These variations gradually decreased in individuals living at the Camargue site (*i.e.*, the mean change in ΔT2-T1OD was +0.26, with a 95% confident interval (95% CI) from +0.14 to 0.38) compared to those living in the city of Marseille (*i.e.*, +0.05 [0.01 to 0.09]), with intermediate variation observed for those living in Fos-sur-mer (*i.e.*, +0.13 [0.07 to 0.18]). The mean changes in ΔT2-T1OD between the Camargue and Fos-sur-mer as well as the Camargue and Marseille sites were significantly different (*p* = 0.019 and *p*<0.0001, respectively, Mann-Whitney test). No significant differences (Mann-Whitney test, ns) were observed when comparing the mean change in ΔT2-T1OD between the Fos-sur-mer and Marseille sites. In contrast, outside of the period of peak exposure to mosquito bites (*i.e.*, T1 and T3) at the three sites, the antibody response against *Aec*SGE returned to the background level at each site (Camargue, +0.04 [−0.06 to 0.14]; Fos-sur-mer, +0.09 [−0.13 to −0.04]; Marseille, +0.01 [−0.02 to 0.04]). Variations in the IgG antibody responses detected between T3 and T1 were considered not to differ (mean ΔT3-T1OD <0.1). The cut-off value for seropositivity (mean aOD ±3 standard deviation) was defined as 1.03 based on the IgG reactivity of sera from individuals living in Marseille who were not exposed to *Ae. caspius*. Individuals showing aOD values above this cut-off level were classified seropositive. Seroprevalence significantly increased among individuals living at the Camargue study site from the T1 time point to the end of the exposure peak (T2), with values increasing from 29% to 54% (chi-squared test, *p*<0.0249, Figure 2B). In addition, seroprevalence significantly declined after the exposure peak and returned to the baseline level (T3 = 29%, chi-squared test, *p*<0.0249). Although an increase in seroprevalence was observed from T1 to T2 and a decrease was observed from T2 to T3 for individuals living at the Fos-sur-mer study site, these variations were not significant (chi-squared test, Figure 2B). No significant change in seroprevalence was observed between T1 and T3 at the Fos-sur-mer and Camargue sites. Additionally, no significant differences were observed when comparing seroprevalence between the two study sites independently of the sampling time (chi-squared test).

### Kinetics of IgG antibody responses to Cx. pipiens salivary gland extracts (CxpSGE) in individuals living in distinct ecological environments

**Figure 3:**
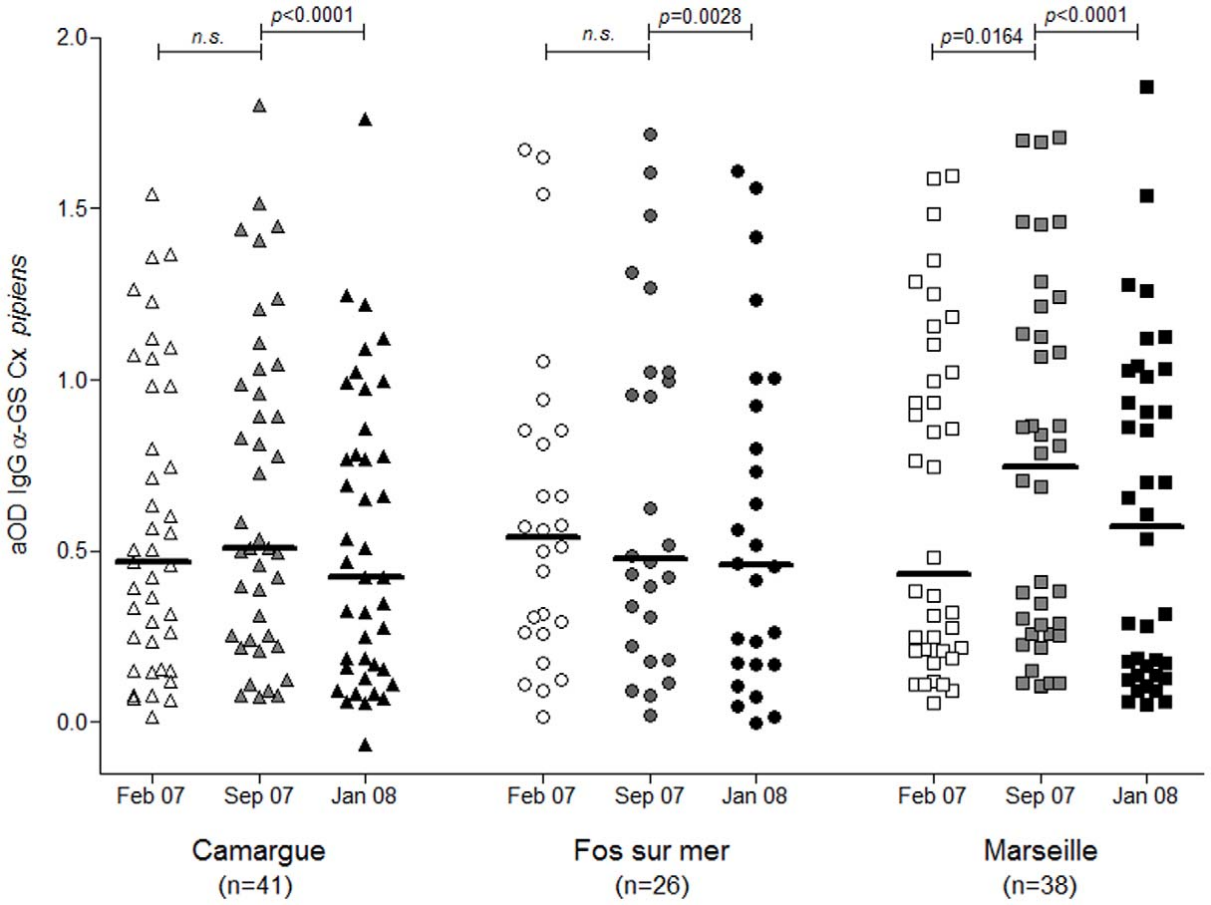
IgG serum levels anti-*Cx. pipiens* salivary gland protein extracts. Scatter plots of IgG antibody responses against *Cx. pipiens* salivary gland extracts in individuals from Marseille (squares), Fos-sur-mer (circles) and Camargue (triangles) at three times, February 07 (T1), September 07 (T2) and January 08 (T3), are represented in white, grey and black symbols, respectively. Antibody responses are represented by aOD: the mean OD value of wells with antigen minus the mean OD value of wells without antigen. Each point shows the aOD value for a single individual. Horizontal bars show medians. Differences between two sampling times at a single site were tested using the Wilcoxon signed-rank test. *p*-values are indicated.

The IgG responses to *CxpSGE* were assessed using the same sera as that used for the *AecSGE* assay. High inter-individual heterogeneity in the antibody responses was observed at all time points and for all sites (Figure 3). Independent of the sampling time (i.e., T1, T2 or T3), no significant difference was observed in the IgG antibody responses to *Cxp*SGE between the sites (Kruskal-Wallis test, Table S1). With respect to the kinetics, despite statistically significant variation being detected in the IgG antibody response to *Cxp*SGE between the T2 and T3 time points for the Camargue and Fos-sur-mer sites (Wilcoxon matched pairs test, *p*<0.0001 and p = 0.0028 for the Camargue and Fos-sur-mer sites, respectively, Table S1), these variations were weak. The IgG antibody variations for the Camargue and Fos-sur-mer sites were considered not to differ (below than 0.1 ΔOD), indicating global stability of IgG responses to *Cxp*SGE throughout the year. Conversely, in the city of Marseille, the variations in the IgG responses observed from T1 to T2 and T2 to T3 were significant (Wilcoxon matched pairs test, *p* = 0.016/74 and p<0.0001 for ΔT2-T1OD and ΔT3-T2OD, respectively) and relevant (ΔT2-T1OD +0.1 [0.01 to 0.19]; ΔT3-T2OD −0.15 [−0.21 to 0.08]).

### Kinetics of antibody responses to Ae. albopictus (AealSGE) and Ae. aegypti salivary gland extracts (AeaeSGE) in individuals living in distinct ecological environments

**Figure 4:**
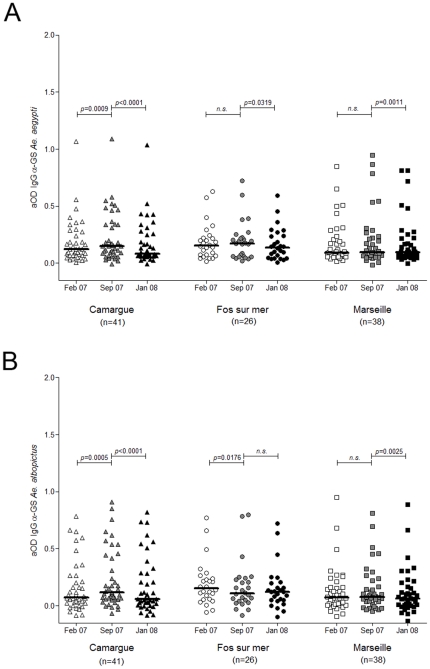
IgG serum levels anti-*Ae. aegypti* and anti-*Ae. albopictus* salivary gland protein extracts. Scatter plots of IgG antibody responses against the salivary gland extracts of *Ae. aegypti* (A) and *Ae. albopictus* (B) in individuals from Marseille (squares), Fos-sur-mer (circles) and Camargue (triangles) at three time points February 07 (T1), September 07 (T2) and January 08 (T3), are represented in white, grey and black symbols, respectively. Antibody responses are represented by aOD: the mean OD value of wells with antigen minus the OD value of wells without antigen. Each point shows the aOD value for a single individual. Horizontal bars show medians. Differences between two time points at a single site were tested using the Wilcoxon signed-rank test. *p*-values are indicated.

To estimate the specificity of the IgG response to *AecSGE*, the same sera were assessed for IgGs against salivary gland extracts of two mosquitoes from the *Aedes* genus (*i.e.*, *Ae. albopictus* and *Ae. aegypti*) that were not endemic in the study area until 2008 (Figure 4) [38]. Independent of the sampling time (*i.e.*, T1, T2 or T3), no significant difference was observed when the IgG antibody responses to *AealSGE* or *AeaeSGE* were compared between sites (Kruskal-Wallis test, Table S1). With regard to the kinetics analysis, despite statistically significant variations being detected in the IgG antibody response to *AealSGE* or *AeaeSGE* between some of the time points at the three sites, these variations were below 0.1 ΔOD and were considered to not differ.

### Correlation of the IgG response between mosquito species

To estimate the level of cross-reactivity of the IgG response to the salivary gland extracts between two mosquito species at T2 (September 2007) at the three sites, a Spearman's rank correlation coefficient (rho) test was used, and the corresponding *p*-values were determined (Table 1). Significant positive correlation coefficients (rho>0.42; *p*<0.0083) were obtained when the mosquitoes of the *Aedes* genus were compared at the three sites, mainly for IgG responses against non-prevalent mosquitoes (*i.e.*, *albopictus* and *aegypti*). Conversely, no significant correlation was observed between the IgG responses against *CxpSGE* and those against the three other *Aedes* species at the three sites, except for the Fos-sur-mer site, where a significant positive correlation coefficient (rho = 0.43; *p* = 0.0079) was obtained when the IgG responses against *CxpSGE* and *AecSGE* were compared (Table 1).

**Table 1:** Correlation of IgG responses between mosquito species.

	*Ae. caspius*	*Ae. albopictus*	*Ae. aegypti*	*Cx. pipiens*
**Camargue site**				
*Ae. caspius*	1			
*Ae. albopictus*	0.4245 (***p*** ** = 0.0046**)	1		
*Ae. aegypti*	0.4526 (***p*** ** = 0.0023**)	0.6187 (***p*** **<0.0001**)	1	
*Cx. pipiens*	0.3168 (*p* = 0.0385)	0.3015 (*p* = 0.0494)	0.2585 (*p* = 0.0942)	1
**Fos-sur-mer site**				
*Ae. caspius*	1			
*Ae. albopictus*	0.5212 (***p*** ** = 0.0004**)	1		
*Ae. aegypti*	0.5529 (***p*** ** = 0.0004**)	0.6529 (***p*** **<0.0001**)	1	
*Cx. pipiens*	0.4300 (***p*** ** = 0.0079**)	0.0842 (*p* = 0.6204)	0.2162 (*p* = 0.1987)	1
**Marseille site**				
*Ae. caspius*	1			
*Ae. albopictus*	0.4648 (***p*** ** = 0.0029**)	1		
*Ae. aegypti*	0.4642 (***p*** ** = 0.0029**)	0.4796 (***p*** ** = 0.0020**)	1	
*Cx. pipiens*	0.1978 (*p* = 0.2274)	0.2513 (*p* = 0.1227)	0.0799 (*p* = 0.6289)	1

Spearman's rank correlation coefficient (rho) and *p*-value between IgG responses against the salivary gland extracts of each pair of mosquito species in September 2007 (T2) (*i.e.*, at the peak of *Ae. caspius* density) are listed. Significant correlations (0.05/6 = 0.0083, *i.e.*, according to the Bonferroni correction for multiple testing) are indicated in bold in brackets.

## Discussion

Numerous studies have reported that mosquitoes' salivary components can induce an antibody response in humans under natural conditions [21], [26], [27], [39]. Here, we analysed human antibody responses against *AecSGE* according to spatial (environment) and temporal (seasons) variations in the level of *Ae. caspius* mosquito exposure. The specificity of the IgG response was also estimated at the genus and species levels.

The Mediterranean coast of southern France includes areas with distinct demographic and ecological conditions that greatly influence the dispersion and composition of the mosquito fauna. Thus, three sites were selected in this area on the basis of environmental patterns influencing *Ae. caspius* density: the Camargue area, the town of Fos-sur-mer and the city of Marseille. In the Camargue wetlands, *Ae. caspius* is well adapted to the rural and swampy environment where it encounters favourable climatic and biotope conditions [34], [35]. The city of Marseille presents an urban habitat that is more suitable for *Cx. pipiens* mosquitoes than rural mosquitoes, such as *Ae. caspius* [35], [40]. Fos-sur-mer, a mid-sized town located between the Camargue area and Marseille, has an intermediary environment and is also exposed to *Ae. caspius* mosquitoes. To confirm the geographic distribution and the different densities of *Ae. caspius* among these three sites, collection of mosquitoes was conducted in July 2007 using carbon dioxide dry ice traps. The collected specimens indicated a decreasing mosquito density gradient from Camargue to Marseille. *Cx. pipiens* and *Ae. caspius* were the most abundant mosquitoes at Camargue (31% and 29%, respectively), as previously described [36], [41]. *Ae. caspius* mosquitoes were captured at the Fos-sur-mer site (21%), but none were found in Marseille (Table S2). Thus, Camargue, Fos-sur-mer and Marseille were considered sites with high, medium and very low levels of exposure to *Ae. caspius* bites, respectively.

In the present study, we showed that the IgG antibody responses to *AecSGE* evolved in accordance with the *Ae. caspius* density, which is influenced by both seasonal changes and the ecological environment. The variations in the IgG antibody levels against *AecSGE* between the peak exposure at T2 and the T1 baseline level were approximately 2- and 4-fold higher in Camargue than in Fos-sur-mer and Marseille, respectively. This positive relationship between anti-salivary protein IgG levels and the seasonal variation of human exposure to mosquito bites has previously been reported [20], [25], [42], [43]. Moreover, the average IgG response level against *AecSGE* observed at T3 returned to T1 baseline levels only four months after the exposure peak (T2), suggesting a short-lived IgG response. A decrease in the IgG response against salivary gland extracts after a period of non-exposure has been described for outdoor workers exposed to ticks [44] and for travellers transiently exposed to *An. gambiae* and *Ae. aegypti* mosquitoes [28]. The transient anti-saliva IgG response and its relationship with mosquito density may be useful for assessing mosquito exposure and could thus, provide new immunological tools to evaluate anti-vector strategies or to monitor vector populations [23]. Recently, Drame and colleagues have confirmed the potential of *An. gambiae* saliva for use as an immunological exposure marker to assess the risk of malaria transmission and the efficiency of antivectorial strategies in a malaria-endemic area [45].

It is worth pointing out that baseline IgG levels against *AecSGE* were significantly and pertinently higher at Camargue and Fos-sur-mer than at Marseille. Repeated seasonal exposure to *Ae. caspius* seemed to favour maintaining a high baseline IgG level throughout the year. To test this hypothesis, a comparison of the kinetics of the IgG response against *AecSGE*, collected outside the *Ae. caspius* exposure peak (cold season) between individuals who had lived for a long period (*i.e.*, at least 5 years) in Camargue and newcomers could be performed. Further studies may also analyse the kinetics of the antibody response in children born in Camargue. Collectively, these data suggest that to determine the prevalence of seroreactivity against *AecSGE*, several parameters should be considered, including mosquito density and the environment, historical mosquito exposure (time spent in a particular area) and individual behaviour (*e.g.*, outdoor/indoor activities, use of mosquito nets or repellents).

To evaluate the specificity of this IgG antibody response, the same sera were first tested against *CxpSGE*. The mosquito collection performed in July 2007 indicated that *Cx. pipiens* was present at all sites, with decreasing densities observed from Camargue to Marseille. In contrast to the IgG response observed to *AecSGE*, the IgG levels against *CxpSGE* were considered not to differ spatially and temporally, with the exception of the levels from Marseille. Individuals from Marseille presented a significant and pertinent increase in the IgG levels against *CxpSGE* after the peak of exposure. These results indicated that unlike the responses against *Ae. caspius*, the IgG responses against *CxpSGE* appeared to not be associated with the decreasing density of *Cx. pipiens* from Camargue to Marseille. This phenomenon could be attributed to a distinct *Cx. pipiens* behaviour that could occur between sites. The temperate species *Culex pipiens* Linné can effectively be divided into two different biological forms: *Culex pipiens pipiens* (*Cx. p. pipiens*) and *Culex pipiens molestus* (*Cx. p. molestus*) [46], [47]. These two subspecies are relatively morphologically similar but exhibit different physiological and behavioural traits [48], [49]. In contrast to the rural *Cx. p. pipiens*, the urban *Cx. p. molestus* is anthropophilic [49], breeds in underground urban habitats, is able to lay its first batch of eggs without a blood meal, does not hibernate and can mate in confined spaces [41], [47]. In Camargue, *Culex pipiens* L. mosquitoes were found at a high density in bird-baited traps compared to horse or human baited-traps, suggesting that the non-anthropophilic form of *Culex pipiens* L. dominates in this rural area [36], [41]. This species is a moderately efficient laboratory West Nile virus vector, but in southern France, it is considered to be a main vector in the dry areas and a secondary vector in the wetlands such as Camargue [36], [41]. Although few entomological data are available for Marseille, the anthropophilic *Cx. p. molestus* form is very likely to occur there, which could represent an explanation for the pertinent IgG increase observed during the warm season. Nevertheless, continuous exposure or an insufficiently long period of non-exposure to *Cx. pipiens* bites throughout the year could limit the IgG baseline feedback, potentially explaining the moderate seasonal variations of IgG that have been observed [49], [50]. Collectively, these data showed that IgG antibody responses against *AecSGE* and *CxpSGE* evolved differently according to site and season, suggesting a specificity of the serological response against *AecSGE*.

Cross-reactivity was evaluated using correlation tests between the IgG levels against *AecSGE* and *CxpSGE* at the exposure peak. Differences were detected in the results according to the location. For Camargue and Marseille, the absence of a significant correlation between the IgG levels against *AecSGE* and *CxpSGE* corresponded to the low antigen cross-reactivity between these two species, which belong to different genera. Conversely, for Fos-sur-mer, a significant positive correlation was detected between these two species. Because both species are present at this site, the correlation could be due more to dual exposure to *Ae. caspius* and *Cx. pipiens* than to antigen cross-reactivity. Thus, our results support a genus-specific IgG response.

It is of note that the IgG response heterogeneity (*i.e.*, between individuals from the same area) observed for *Cx. pipiens* and, to a lesser extent, for *Ae. caspius* might reflect heterogeneous exposure to mosquito bites due to individual behaviours (*e.g.*, outdoor/indoor activities, use of mosquito nets or repellents). Additionally, these two mosquito species exhibit distinct circadian biting activities (*e.g.*, clearly diurnal and nocturnal biting activities are observed for *Ae. caspius* and *Cx. pipiens* mosquitoes, respectively) [36], which could further increase this inter-individual heterogeneity.

Finally, intra-genus specificity was estimated using SGE from two *Aedes* species that were not endemic at the three sites during the time of the study. The very low IgG levels observed against *AealSGE* and *AeaeSGE*, independent of the site and timing, indicated that the IgG responses to *AecSGE* were specific at the species level. Nevertheless, the significant correlation coefficients obtained when the levels of IgG against mosquitoes from the *Aedes* genus were compared suggest cross-reactivity. These correlations were not attributed to dual exposure (because these mosquitoes were not present in the study area), but to the presence of shared salivary antigens between different *Aedes* species [51].

Collectively, these data showed that the IgG antibody response against *AecSGE* may be related to seasonal and geographical variations in *Ae. caspius* density. The pertinent increase and transient IgG response at the peak of exposure appears to be species-specific, and these results strongly suggest that human antibody responses may be used to assess the individual level of exposure to mosquito bites. Nevertheless, other parameters should be considered, including historical individual exposure, which could influence the baseline IgG level. Further studies are needed to characterise specific *Aec*SG antigens, for instance, using an immunoproteomic approach, as described previously [52]–[54]. This step of identifying the antigenic protein repertoire is necessary to determine the diversity and specificity of this repertoire. Salivary proteins of different arthropod species can share sequence similarities [55] and cross-reacting antigens [56], [57], resulting in the need to select species-specific antigens. Recombinant forms of selected salivary gland antigen candidates could be used for the development of a more sensitive and specific immunological test to accurately assess individual exposure to mosquito bites. Thus, specific immune responses against mosquito saliva antigens could be used in control and surveillance programs to assess the efficiency of anti-mosquito strategies, to estimate exposure levels and to identify new infestation areas. This strategy could be extended to other mosquito species that are involved in the transmission of infectious diseases and could represent a tool for estimating the risk of vector-borne disease transmission. Collectively, these data confirm that human antibody responses may be used to assess individual exposure to mosquito bites from particular species and estimate the level of these pests.

## Materials and Methods

### Ethics statement

All participants gave their written informed consent to participate in the study, and the Marseille-2 Ethical Committee approved the protocol (N°2006-A00581-50). Mosquito larval sampling was carried out in non-privately owned areas and non-protected areas outside the boundaries of the regional nature park of Camargue. The field study did not involve endangered or protected species. No specific permissions were required for the described field studies.

### Study sites

The study was conducted in the Provence-Alpes-Côte d'Azur (PACA) area in southeastern France. Three study sites in PACA were chosen: (i) Camargue, a large wetland area of 150,000 hectares [35] located inside the Rhone River delta, principally covered with pools of water, marshes and irrigated fields, where the human population is distributed between towns, hamlets and isolated houses [58], [59]; (ii) Fos-sur-mer, a town with 14,000 inhabitants (population density, 151 inhabitants/km^2^) with a mixed residential and agricultural landscape, located approximately 15 km from the border of the Camargue area; and (iii) the city of Marseille, a dry, an urban area with approximately 852 400 inhabitants and located approximately 30 km from Fos-sur-mer (Figure 1).

### Studied population

Volunteers were recruited from Camargue (n = 41, 54% male, mean age ± SD: 45.7±11.3, Caucasian), Fos-sur-mer (n = 26, 42% male, mean age± SD: 51.5±11, Caucasian) and Marseille (n = 38, 47% male, mean age± SD: 40.3±12.2, Caucasian). For each individual, blood samples were collected by venous puncture at three different time points: February 2007, September 2007 and January 2008. Sera were obtained through centrifugation of the blood samples and were stored at −20°C. Eligible participants were individuals who did not travel to countries or areas that are endemic for *Ae. aegypti* and *Ae. albopictus* mosquitoes in the six months prior to and during the study [60]–[62].

### Mosquitoes and salivary gland extraction

Adult female *Ae. caspius*, *Cx pipiens*, *Ae. aegytpi* and *Ae. albopictus* mosquitoes were used in this study. *Ae. caspius* and *Cx. pipiens* species were collected at the larval stage in the field in Camargue from August to September 2009, and the mosquitoes were reared in an insectarium. The *Ae. albopictus* mosquito colony came from the Alpes-Maritimes area and was bred in a laboratory at the Entente Interdépartementale pour la Démoustication (EID) Méditerranée (Cagnes-sur-Mer). *Ae. aegypti* mosquitoes came from the Bora-Bora reference colony, which was bred in a laboratory at the Institut de Recherche pour le Développement (Montpellier). All of these mosquitoes were maintained under identical standard conditions of 26°C and 60% humidity. All mosquitoes consumed no blood meals and were maintained on a diet of a 10% syrup solution. Salivary glands from 5- to 8-day old adult female mosquitoes were dissected on ice in phosphate-buffered saline (PBS) under a stereomicroscope. The salivary glands were pooled by species in a microcentrifuge tube and were then stored frozen at −20°C until needed. At that time, the salivary glands were disrupted by ultrasonication (Vibracell 72412, Bioblock Scientific, Illkirch, France) for 5 min on ice at maximum amplitude. Salivary gland homogenates were then centrifuged for 15 min at 16,100× g [63] and the protein concentration of the supernatant was determined in duplicate by the Lowry method (DC Protein assay Kit, Bio-Rad) according to the manufacturer's instructions. Salivary gland proteins were then suspended in 0.1 M (pH 9.6) bicarbonate buffer to obtain a protein concentration of 1 µg/µL.

### ELISA

The sera were tested by ELISA for the presence of IgG antibodies that bind to salivary gland proteins. To optimise the working conditions of the ELISA tests, a checkerboard titration was performed to establish salivary gland protein extracts and serum conditions. Based on the results of this procedure, Maxisorp Microtiter Immunoplates (Nunc, Denmark) were coated with 2 µg/ml (50 µl/well) of either *Ae. caspius*, *Cx. pipiens*, *Ae. albopictus* or *Ae. aegypti* salivary gland extracts diluted in 0.1 M bicarbonate buffer (pH 9.6) overnight at 4°C. Three washes were performed with 250 µL of PBS (pH 7.4, Sigma Co., USA) plus 0.05% Tween-20 (Sigma Co., USA) between each incubation. The plates were blocked for 2 h at 37°C with 200 µL of blocking solution buffer consisting of PBS, 0.05% Tween and 5% skimmed milk (Beckton, Dickinson Bioscience, USA). Serum diluted 1∶50 in blocking buffer was added (50 µl/well) to the plates, and they were incubated at 37°C for 1 h [64]. Subsequently, 50 µl of horse radish peroxidase (HRP)-conjugated rabbit anti-human IgG (1∶10,000, Invitrogen, Rockville, USA) diluted in the blocking buffer were added, followed by incubation for 1 h at 37°C. Enzyme activity was detected by incubation with 50 µl of tetramethylbenzidine substrate (KPL, USA) for 10 min at room temperature. The reaction was stopped using 50 µl of 1 M H_2_SO_4_. The optical density (OD) at 450 nm was determined with a microplate reader (Versa Max® Turnable Multiplate Reader, Molecular Devices, UK). Each serum sample was tested in duplicate and in control wells without salivary gland extracts. To improve the consistency of the results, sera from different study sites were randomly arranged on each plate, and the samples collected at different time points for each individual were tested on the same plate. A pool of 5 sera collected in September 2007 from individuals living in Camargue (selected based on ELISA optimisation tests) was used as a positive control on all plates coated with salivary gland extracts from *Ae. caspius* and *Cx. Pipiens*. A pool of 5 sera from individuals living in inter-tropical areas, kindly provided by Dr. F. Remoué, was used as a positive control against salivary gland extracts from *Ae. aegypti* [25] and *Ae. albopictus*. Only plates presenting inter-assay variations in absorbance values of positive controls lower than 20% were included in the analysis. The levels of IgG antibodies were expressed as the adjusted OD (aOD), which was calculated for each serum sample as the mean OD value for wells with salivary gland extracts minus the OD value of the control wells, i.e., without salivary gland extracts. Individual variations in IgG antibody responses were assessed according to the OD differences (ΔOD) between pairs of sera collected throughout the year. To consider pertinent ΔOD between pairs of sera, an arbitrary threshold of 0.1 ΔOD was defined [65]. The mean aOD at the three time points for individuals not exposed to *Ae. caspius* living in Marseille plus 3 standard deviations was used as the cut-off value for seropositivity.

### Statistical analyses

After verifying that the values in each group did not assume a Gaussian distribution, the Kruskal-Wallis, Mann-Whitney and Wilcoxon matched-pairs signed-rank tests and Spearman's rank correlation coefficient were computed when appropriate with STATA version 9.0 (Stata-Corp, USA). The frequencies were compared by the chi-squared test. All differences were considered significant at *p*<0.05. However, for multiple tests, a Bonferroni correction was applied and *p*-value significance was then indicated.

## Supporting Information

Table S1 **Statistical analysis of spatial and temporal variations in IgG responses.** The Kruskal-Wallis tests were used to compare the antibody levels between more than two independent groups (geographical comparisons). Wilcoxon matched-pairs signed-rank tests were used to compare paired sera between two time points. All significant differences (*p*<0.05) are indicated in bold. SD: standard deviation, aOD: adjusted optical density, CI: confident interval, T1: February 07, T2: September 07, T3: January 08.
(DOC)

Table S2 **Adult mosquitoes captured at each site in July 2007 using carbon dioxide dry ice traps.** Carbon dioxide traps were hung in 5 locations in each study site during 24 hrs. Mosquitoes were identified using morphological characteristics and identification keys. The mean number of mosquitoes sampled in each site was calculated using the results of the five traps. The proportion of each mosquito genus/species per site is indicated into brackets.
(DOC)
