# Supplementary material for: Relationship between Exposure to Vector Bites and Antibody Responses to Mosquito Salivary Gland Extracts
Source: PLoS One. 2011 Dec 14;6(12):e29107. doi: 10.1371/journal.pone.0029107 (PMC3237593; doi:10.1371/journal.pone.0029107)
Supplement: Table S1 — Statistical analysis of spatial and temporal variations in IgG responses. The Kruskal-Wallis tests were used to compare the antibody levels between more than two independent groups (geographical comparisons). Wilcoxon matched-pairs signed-rank tests were used to compare paired sera between two time points. All significant differences (p<0.05) are indicated in bold. SD: standard deviation, aOD: adjusted optical density, CI: confident interval, T1: February 07, T2: September 07, T3: January 08. (DOC) [file pone.0029107.s001.doc]

**Table S1**

|  |  | **Camargue (*n=41*)** | | | | **Fos-sur-mer (*n=26*)** | | | | **Marseille (*n=38*)** | | | | *p-value* |
| --- | --- | --- | --- | --- | --- | --- | --- | --- | --- | --- | --- | --- | --- | --- |
|  |  |  |  | 95% CI | |  |  | 95% CI | |  |  | 95% CI | | (Kruskal-Wallis) |
|  | Time | Mean | SD | Lower | Upper | Mean | SD | Lower | Upper | Mean | SD | Lower | Upper |  |
| ***Ae. caspius* (aOD)** |  |  |  |  |  |  |  |  |  |  |  |  |  |  |
|  | T1 | 0.81 | 0.38 | 0.69 | 0.92 | 0.74 | 0.32 | 0.61 | 0.87 | 0.35 | 0.21 | 0.28 | 0.42 | ***< 0.0001*** |
|  | T2 | 1.07 | 0.39 | 0.94 | 1.19 | 0.86 | 0.35 | 0.72 | 1.00 | 0.41 | 0.25 | 0.33 | 0.49 | ***< 0.0001*** |
|  | T3 | 0.84 | 0.37 | 0.73 | 0.96 | 0.65 | 0.32 | 0.52 | 0.78 | 0.35 | 0.20 | 0.29 | 0.42 | ***< 0.0001*** |
| *p-value* | T1 vs. T2 | ***< 0.0001*** |  |  |  | ***0.0018*** |  |  |  | ***< 0.0001*** |  |  |  |  |
| (Wilcox. signed-rank test) | T2 vs. T3 | ***< 0.0001*** |  |  |  | ***< 0.0001*** |  |  |  | ***< 0.0001*** |  |  |  |  |
|  | T1 vs. T3 | *0.3537* |  |  |  | ***0.0007*** |  |  |  | *0.9422* |  |  |  |  |
| ***Cx. pipiens* (aOD)** |  |  |  |  |  |  |  |  |  |  |  |  |  |  |
|  | T1 | 0.57 | 0.43 | 0.44 | 0.71 | 0.61 | 0.47 | 0.42 | 0.80 | 0.64 | 0.49 | 0.48 | 0.81 | *0.9158* |
|  | T2 | 0.66 | 0.47 | 0.51 | 0.81 | 0.66 | 0.51 | 0.46 | 0.87 | 0.74 | 0.52 | 0.57 | 0.91 | *0.7851* |
|  | T3 | 0.53 | 0.43 | 0.40 | 0.66 | 0.57 | 0.49 | 0.37 | 0.77 | 0.60 | 0.49 | 0.44 | 0.76 | *0.8662* |
| *p-value* | T1 vs. T2 | *0.063* |  |  |  | *0.509* |  |  |  | ***0.0174*** |  |  |  |  |
| (Wilcox. signed-rank test) | T2 vs. T3 | ***< 0.0001*** |  |  |  | ***0.0028*** |  |  |  | ***< 0.0001*** |  |  |  |  |
|  | T1 vs. T3 | *0.0519* |  |  |  | *0.2326* |  |  |  | ***0.0255*** |  |  |  |  |
| ***Ae. albopictus* (aOD)** |  |  |  |  |  |  |  |  |  |  |  |  |  |  |
|  | T1 | 0.16 | 0.22 | 0.09 | 0.23 | 0.19 | 0.20 | 0.12 | 0.27 | 0.13 | 0.20 | 0.06 | 0.20 | *0.153* |
|  | T2 | 0.23 | 0.26 | 0.15 | 0.31 | 0.17 | 0.22 | 0.08 | 0.26 | 0.15 | 0.20 | 0.08 | 0.22 | *0.3403* |
|  | T3 | 0.17 | 0.25 | 0.10 | 0.25 | 0.15 | 0.19 | 0.08 | 0.23 | 0.12 | 0.20 | 0.06 | 0.19 | *0.5195* |
| *p-value* | T1 vs. T2 | ***0.0005*** |  |  |  | ***0.0182*** |  |  |  | *0.149* |  |  |  |  |
| (Wilcox. signed-rank test) | T2 vs. T3 | ***< 0.0001*** |  |  |  | *0.2087* |  |  |  | ***0.0028*** |  |  |  |  |
|  | T1 vs. T3 | *0.5004* |  |  |  | ***0.0003*** |  |  |  | *0.2875* |  |  |  |  |
| ***Ae. aegypti* (aOD)** |  |  |  |  |  |  |  |  |  |  |  |  |  |  |
|  | T1 | 0.18 | 0.19 | 0.12 | 0.24 | 0.19 | 0.15 | 0.13 | 0.25 | 0.18 | 0.19 | 0.12 | 0.24 | *0.6958* |
|  | T2 | 0.23 | 0.22 | 0.16 | 0.29 | 0.20 | 0.17 | 0.13 | 0.26 | 0.20 | 0.23 | 0.12 | 0.27 | *0.5416* |
|  | T3 | 0.17 | 0.19 | 0.11 | 0.23 | 0.17 | 0.14 | 0.11 | 0.23 | 0.18 | 0.21 | 0.11 | 0.25 | *0.9002* |
| *p-value* | T1 vs. T2 | ***0.001*** |  |  |  | *0.6115* |  |  |  | *0.5401* |  |  |  |  |
| (Wilcox. signed-rank test) | T2 vs. T3 | ***< 0.0001*** |  |  |  | ***0.0324*** |  |  |  | ***0.0011*** |  |  |  |  |
|  | T1 vs. T3 | *0.123* |  |  |  | ***0.0289*** |  |  |  | *0.1845* |  |  |  |  |
|  |  |  |  |  |  |  |  |  |  |  |  |  |  |  |
