# Supplementary material for: Relationship between Exposure to Vector Bites and Antibody Responses to Mosquito Salivary Gland Extracts
Source: PLoS One. 2011 Dec 14;6(12):e29107. doi: 10.1371/journal.pone.0029107 (PMC3237593; doi:10.1371/journal.pone.0029107)
Supplement: Table S2 — Adult mosquitoes captured at each site in July 2007 using carbon dioxide dry ice traps. Carbon dioxide traps were hung in 5 locations in each study site during 24 hrs. Mosquitoes were identified using morphological characteristics and identification keys. The mean number of mosquitoes sampled in each site was calculated using the results of the five traps. The proportion of each mosquito genus/species per site is indicated into brackets. (DOC) [file pone.0029107.s002.doc]

**Table S2**

| **Mosquito spp.** | **Camargue** | **Fos-sur-mer** | **Marseille** |
| --- | --- | --- | --- |
|  | **(%)** | **(%)** | **(%)** |
| *Aedes* | 1512 (54.2) | 37 (22.4) | 0 |
| *Ae. caspius* | 798 (28.6) | 34 (20.6) | 0 |
| *Ae. aegypti* | 0 | 0 | 0 |
| *Ae. albopictus* | 0 | 0 | 0 |
| *Other Aedes* | 714 (25.6) | 3 (1.8) | 0 |
| *Culex* | 937 (33.6) | 120 (72.7) | 25 (78.1) |
| *Cx. pipiens* | 858 (30.8) | 120 (72.7) | 25 (78.1) |
| *Other Culex* | 79 (2.8) | 0 | 0 |
| *Anopheles* | 339 (12.2) | 1 (0.6) | 0 |
| *Culiseta* | 0 | 7 (4.2) | 7 (21.9) |
